# TNMB分期在肺癌中的研究进展

**DOI:** 10.3779/j.issn.1009-3419.2025.106.28

**Published:** 2025-10-20

**Authors:** Yuanyuan ZHAN, Yue LI, Cheng SHEN

**Affiliations:** ^1^610041 成都，四川大学华西医院临床医学院（詹媛媛，李悦）; ^1^West China School of Medicine; ^2^胸外科（沈诚）; ^2^Department of Thoracic Surgery, West China Hospital, Sichuan University, Chengdu 610041, China

**Keywords:** TNMB分期, TNM分期, 肺肿瘤, 分子标志物, TNMB staging, TNM staging, Lung neoplasms, Molecular markers

## Abstract

肺癌是我国发病率和死亡率均居首位的恶性肿瘤，准确的临床分期对其预后判断和治疗策略的制定至关重要。目前国际公认的肿瘤原发灶-区域淋巴结-远处转移（tumor-nodule-metastasis, TNM）分期系统以肿瘤解剖学特征为核心，但难以反映肿瘤生物学本质。TNM-血液受累（tumor-nodule-metastasis-blood, TNMB）分期在TNM分期的基础上融入血液分子标志物，实现了解剖学与生物学特征的多维度评估，最初应用于皮肤T细胞淋巴瘤，近年逐渐拓展至肺癌领域。本文综述TNMB分期在肺癌中的现有研究应用，对比分析其与传统TNM分期在分期依据、评估维度及临床效能上的差异，并进一步总结了现有B分期相关分子标志物类型及有望纳入B分期的分子标志物。

## Abstract (en)

Lung cancer is the malignant tumor with the highest incidence and mortality in China, and accurate clinical staging is crucial for judging its prognosis and formulating treatment strategies. Currently, the internationally recognized tumor-nodule-metastasis (TNM) staging system centers on tumor anatomical characteristics but struggles to reflect the biological essence of tumors. Based on the TNM staging, the TNM-blood (TNMB) staging incorporates blood molecular biomarkers to achieve multi-dimensional assessment of anatomical and biological characteristics. Initially applied to cutaneous T-cell lymphoma, it has gradually expanded to the field of lung cancer in recent years. This review summarizes the existing research and applications of TNMB staging in lung cancer, comparatively analyzes the differences between TNMB and the traditional TNM staging in terms of staging basis, evaluation dimensions and clinical efficacy, and further summarizes the existing types of molecular biomarkers related to B staging as well as those expected to be included in B staging.

肺癌是起源于肺部支气管黏膜或腺体的恶性肿瘤，在男性群体中更为高发。在我国，肺癌居癌症发病谱和死因谱的第1位，标化发病率与死亡率均高于世界水平，其预后情况的判断和治疗策略的制定，均高度依赖于准确的临床分期^[1]^。目前，国际上公认的肺癌分期标准是肿瘤原发灶-区域淋巴结-远处转移（tumor-nodule-metastasis, TNM）分期系统。近年来，随着分子生物学技术的飞速发展，研究者们^[2]^发现血液中的分子标志物在肺癌分期中具有一定的补充价值。基于此，在传统TNM分期的基础上，融入血液中的分子标志物，形成了TNM-血液受累（TNM-blood, TNMB）分期系统。本文对TNMB分期系统在肺癌中的应用及其在分期中的优势进行综述。

## 1 传统TNM分期与TNMB分期

TNM分期系统由法国医生Pierre Denoix于20世纪40年代提出，1968年国际抗癌联盟（Union for Inter-national Cancer Control, UICC）正式发布第1版《恶性肿瘤TNM分类》^[3]^，随后与美国癌症联合委员会（American Joint Committee on Cancer, AJCC）合作，每6-8年更新一次。目前临床上使用最为广泛的是UICC于2017年颁布的第8版TNM分期，该版TNM分期依旧是以肿瘤解剖学特征为核心，通过原发肿瘤大小、区域淋巴结转移和远处转移划分疾病进展程度。相较于第7版，第8版TNM分期进一步细化了T分期、调整了M分期并优化了分期组合，旨在更精准地反映不同分期患者的预后差异^[4]^。2024年，第9版肺癌TNM分期发布，相较于第8版，其重要变化包括对部分分期亚型的调整、扩展病例来源和将分子标志物纳入考虑^[5,6]^（表1）。其中纳入分子标志物这一点与TNMB分期理念相似，但第9版TNM分期仅提出这一概念，并未有具体的实施标准，使其核心理念仍然局限于解剖学维度。

**表1:** 第9版TNM分期较第8版有所改动的内容

Item	Modified content	Clinical significance
T-stage	No changes	-
N-stage	Subdividing N2 stage into N2a and N2b, representing unilaterally single-site and multi-site mediastinal lymph node metastasis, respectively	Distinguishing the disease heterogeneity among N2-stage patients, assessing disease status and prognosis, and formulating treatment plans
M-stage	Stage M1c is subdivided into stages M1c1 and M1c2, respectively representing multiple metastases to a single organ outside the chest cavity and multiple metastases to multiple organs	Reflects the prognostic differences associated with different distant metastasis patterns
Stage I	No changes	Avoid overtreatment or undertreatment, and some patients can gain access to surgical options
Stage II	1) T1N1M0 downgraded from IIB to IIA 2) T1N2aM0 downgraded from IIIA to IIB	
Stage III	1) T1N2bM0 upgraded from IIIA to IIIB 2) T3N2aM0 downgraded from IIIB to IIIA	
Stage IV	No changes	

TNM: tumor-nodule-metastasis.

TNMB分期最早由国际皮肤淋巴瘤学会（International Society for Cutaneous Lymphomas, ISCL）与欧洲癌症研究与治疗组织（European Organization for Research and Treatment of Cancer, EORTC）联合提出，最初用于蕈样肉芽肿和Sézary综合征的分期，且在该分期体系中首次纳入了血液受累（B）这一指标。随着生物学特征在肿瘤治疗中的重要性日益凸显，TNMB分期的概念也逐渐拓展应用到其他癌症领域^[7]^。在当前肺癌相关研究领域，研究者多先依据循环肿瘤DNA（circulating tumor DNA, ctDNA）、特定基因表达谱等分子指标，对患者进行独立的风险分层；随后再以该风险分层标准为依据，对传统TNM分期结果进行调整，例如，Kratz等^[8]^和Haro等^[9]^的研究便是基于特定基因表达谱构建风险分级体系，据此将TNM分期结果上调或下调一级；另有研究则通过二者的深度结合形成全新TNMB分期，如Chen等^[10]^的团队通过整合微小残留病灶（minimal residual disease, MRD）的阴性/阳性状态与TNM I-III期分期结果，最终确立了TNMB I-III期分期标准。总体而言，TNMB分期能够从多个维度全面地解析肿瘤的生物学特征，为临床评估提供更丰富的信息支撑。以淋巴瘤领域为例，TNMB分期在皮肤T细胞淋巴瘤的诊疗实践中呈现出显著优势。该分期系统通过T、N、M、B四个维度的综合评估，能够精准界定患者的疾病严重程度，是判断生存预后及进展风险的核心指标。具体而言，T纬度依据皮肤受累面积（是否>10%体表面积）和病变类型（斑片、斑块、肿瘤、红皮病）划分，N纬度关注淋巴结受累程度，M纬度评估内脏转移情况，B纬度则依据异常T细胞数量来判断血液受累程度，通过这样的多纬度评估，患者被明确分为IA-IVB期，各期对应的中位总生存期（overall survival, OS）、10年OS率和疾病进展风险差异显著，例如IA期患者10年OS率达88%，而IVB期患者5年OS率仅为18%。同时，其为“风险适配”治疗策略的制定提供了可靠依据：对于局限于皮肤的早期患者（如IA、IB期），优先采用皮肤定向治疗；对于存在显著淋巴结、内脏、血液受累的晚期患者（如IIB期及以上），以全身治疗为主，必要时可考虑干细胞移植。这一模式提升了早、晚期患者治疗方案的精准性，有效规避了过度治疗或治疗不足的情况，充分彰显了TNMB分期在肿瘤分期体系中的独特价值^[11,12]^。

## 2 TNMB分期在肺癌分期中的现有应用

既往TNMB分期系统的应用主要局限于皮肤T细胞淋巴瘤，近年来，已有学者开始尝试将TNMB分期引入肺癌分期领域，探索其在肺癌病情评估中的潜在价值。下文将依次梳理目前相关研究的核心内容，且相关研究的研究设计类型、所采用的关键分子生物学技术及研究结果总结见表2^[8-10,13]^。

**表2:** TNMB分期在肺癌中的相关研究及核心信息汇总

Number	Research	Type	Molecular biology techniques	TNMB stage	Result
1	Incorporation of a molecular prognostic classifier improves conventional NSCLC staging^[8]^	Retrospective cohort study	14-gene molecular prognostic classifier (11 cancer-related target genes+3 reference genes)	Establish a prognostic model using the 14-gene molecular prognostic classifier for risk scoring, and adjust the patient's TNM staging based on the risk score	Significantly improves identification of high-risk patients and survival prediction compared to the 8^th^ edition TNM staging system, with a net reclassification improvement of 0.33 and a relative overall discrimination improvement of 22.1%
2	Comparison of traditional TNM and novel TNMB staging systems for NSCLC^[9]^	Prospective cohort study	14-gene molecular prognostic classifier (11 cancer-related target genes+3 reference genes)	Establish a prognostic model using the 14-gene molecular prognostic classifier for risk scoring, and adjust the patient's TNM staging based on the risk score	Outperforms traditional TNM staging in DFS estimation, with a net reclassification improvement of 0.28 and enhanced model fit and discriminatory power
3	Individualized tumor-informed ctDNA analysis for postoperative monitoring of NSCLC^[10]^	Prospective cohort study	Detect ctDNA in plasma using PROPHET technology to achieve highly sensitive monitoring of MRD status	TNMB=TNM+MRD: TNMB I=TNM I+MRD (-) TNMB II=TNM II/III+MRD (-) TNMB III=TNM I-III+MRD(+)	PROPHET technology demonstrates high sensitivity in MRD status detection; MRD status is a predictive factor for postoperative outcomes in NSCLC; TNMB staging systems incorporating MRD status outperform traditional TNM staging in postoperative prognosis prediction
4	Genomic profiling of aggressive pathologic features in lung adenocarcinoma^[13]^	Retrospective cohort study	NGS, utilizing four distinct panels comprising 520, 168, 196, and 139 cancer-associated mutation genes respectively	Key mutations serve as a potential indicator for survival prediction in TNMB staging, though the specific TNMB staging criteria are not detailed in the article	The association between multiple pathological features and driver mutations has been clarified. Exclusive mutations in *STK11*, *PTEN*, and *TOP2A* may serve as potential predictors for TNMB classification. TNMB staging demonstrates superior performance in prognostic evaluation

TNMB: tumor-node-metastasis-blood; MRD: minimal residual disease; NSCLC: non-small cell lung cancer; NGS: next-generation sequencing; STK11: serine/threonine kinase 11; PTEN: phosphatase and tensin homolog; TOP2A: DNA topoisomerase II alpha; ctDNA: circulating tumor DNA; DFS: disease-free survival.

2019年Kratz等^[8]^进行了一项回顾性研究，旨在探究将分子预后分类器整合到非小细胞肺癌（non-small cell lung cancer, NSCLC）分期中的效果，研究者将14基因分子预后分类器整合到第8版TNM分期系统中，开发出了一套TNMB分期系统。该14基因分子预后分类器涉及的基因多为经典致癌通路中的关键基因，研究团队通过检测目标基因的表达量构建预后模型，对队列中的患者进行风险评分，并根据风险评分，按33%和67%分位数的临界值将患者划分为低、中、高3个风险等级，以此风险分级标准作为模型预测患者预后的依据。该14基因分子预后分类器的准确性与有效性，在此前已有相关研究^[14]^进行过验证与评估。该TNMB分期系统则是沿用了第8版NSCLC TNM分期系统的顺序和优先级，但允许根据预后模型的风险评分进行调整，复发高风险患者的分期上调一级，复发低风险患者的分期下调一级，复发中风险患者的分期则无变化。该TNMB分期系统的开发最初基于加州大学旧金山分校321例NSCLC患者的数据^[8]^，随后在包含1373例患者的多中心独立队列中进行验证。研究人员将该TNMB系统与第7、8版TNM分期系统进行对比分析，主要采用重新分类指标等评估方法，以OS为主要结局指标。结果显示，与第8版TNM分期相比，TNMB分期系统在识别高危患者方面表现更优，净重新分类改善为0.33（95%CI: 0.24-0.41）；在生存预测上更出色，相对综合鉴别改善达22.1%（95%CI: 8.8%-35.3%），且观测与预测生存的一致性更好，重新分类校准统计量从39降至21。该研究首次将分子预后分类器整合到连贯的肺癌分期系统中，证明了TNMB分期系统在风险分层和生存预测方面显著优于传统TNM分期系统。同时，该团队也开展了一项前瞻性队列研究^[9]^，纳入238例2012-2018年间于加州大学旧金山分校接受手术切除的I-IIIC期非鳞NSCLC患者，同样对患者按照第8版TNM分期系统及TNMB分期系统进行重新分期，运用重新分类统计量评估各系统，以3年无病生存期（disease-free survival, DFS）为主要结局指标。结果显示，66.8%的患者经TNMB系统重新分期；与第7、8版TNM相比，TNMB系统的模型拟合度显著提升[*R*^2^从0.22升至0.31，一致性指数从0.68升至0.73，*Log-rank *χ^2^从38升至108，且净重新分类改善达0.28（95%CI: 0.08-0.46, *P*<0.001）]。该研究进一步证实TNMB分期系统在NSCLC中能更精准预测DFS，增强高危患者识别及复发与非复发患者区分度，为临床决策提供更可靠依据。

2023年Chen等^[10]^开展了一项前瞻性研究，旨在探究个体化肿瘤知情ctDNA分析在NSCLC术后监测中的价值。研究团队开发出了一套个性化的肿瘤知情技术——患者特异性预后及潜在治疗标志物追踪技术（patient-specific prognostic and potential therapeutic marker tracking, PROPHET）。PROPHET技术是基于患者肿瘤组织的全外显子测序（whole exome sequencing, WES）数据，筛选出最多50个患者特异性变异位点，设计相应的个性化捕获panel，通过超高深度靶向测序检测血浆中的ctDNA，实现对肿瘤MRD的高灵敏度检测。研究团队对181例早期NSCLC患者的760份血浆样本分别采用PROPHET技术和现有肿瘤知情固定panel（覆盖168个肺癌相关基因）在术后3-7 d及术后1个月两个时间点进行MRD检测并长期随访患者情况，研究结果表明PROPHET技术对DFS和OS的预测能力均显著优于固定panel，且PROPHET技术中包括的非经典变异位点在MRD检测中贡献显著。该项研究^[10]^与现有其他研究^[15]^均显示，MRD状态是与NSCLC术后预后显著相关的因素。在该项研究中，研究人员分析了PROPHET检测的特定时间点的MRD状态与其他临床病理因素（如TNM分期、肿瘤大小等）对DFS预测的贡献度，发现MRD状态对DFS预测的贡献度高达85.6%。基于这项技术对MRD检测的准确性及MRD状态对NSCLC预后的独特价值，研究团队提出了整合TNM分期与MRD状态的TNMB分期。在该TNMB分期中，患者被分为TNMB I-III期，其中TNMB I期为TNM I期且MRD状态阴性的患者；TNMB II期为TNM II/III期且MRD状态阴性的患者；TNMB III期为TNM I-III期且MRD状态阳性的患者。研究结果表明，整合TNM分期与MRD状态形成的TNMB分期，在预后预测上优于传统TNM分期，纵向MRD检测的阴性预测值在180、360、720 d分别为99.4%、98.4%、95.5%，且MRD动态变化率有助于改善预后预测。这表明TNMB分期在肺癌应用中能提升预后评估准确性，增强高危患者识别能力，为术后监测和治疗决策提供更可靠的依据^[10]^。

2025年Lin的团队^[13]^开展了一项关于肺腺癌侵袭性病理特征的基因组分析研究。该研究纳入1559例腺癌样本进行常见驱动基因突变分析，1306例样本进行基因组图谱分析，通过回顾性分析患者的临床、病理、下一代测序技术（next-generation sequencing, NGS）结果，探究不同病理特征中常见驱动突变的分布，区分不同病理特征中的关键突变和致癌通路改变，并将关键分子事件整合到TNMB分期和预后分析中。该研究表明丝氨酸/苏氨酸激酶11（serine/threonine kinase 11, *STK11*）、第10号染色体缺失的磷酸酶与张力蛋白同源物（phosphatase and tensin homolog deleted on chromosome ten, *PTEN*）和DNA拓扑异构酶IIα（DNA topoisomerase II alpha, *TOP2A*）的排他性突变组合可能是TNMB分类中生存预测的一个潜在指标。该研究团队未写明他们如何将关键分子事件整合到TNMB分期，但在研究中他们对TNMB分期和TNM分期进行了对比，结果显示在TNMB分期中，II与I期相比，风险比（hazard ratio, HR）为2.28（95%CI: 1.36-3.86, *P*<0.001），III与II期相比，HR为1.95（95%CI: 1.04-3.21, *P*=0.031），其不同分期间的HR均高于传统TNM分期。这一方面表明TNMB分期有助于阐明病理特征的侵袭性表型，另一方面体现出TNMB分期在预后评估中可能是一种更有前景的方法，能更精准地评估患者预后，为肺腺癌的分期和预后评估提供了新见解。

TNMB分期在肺癌领域展现出了巨大潜力。诊断方面，其通过整合分子信息，能更精准地进行风险分层；治疗方面，可为临床决策提供可靠依据；预后方面，无论是生存预测的准确性还是预后评估的时效性和动态监测方面，TNMB分期表现均优于传统TNM分期。

## 3 传统TNM分期与TNMB分期在肺癌诊疗中的区别

TNMB分期作为在传统TNM分期基础上发展而来的新型分期系统，二者在核心依据、评估维度、临床价值等方面存在显著差异，这些差异也决定了它们在肺癌诊疗实践中不同的应用场景和效能（表3）。

**表3:** 传统TNM分期与TNMB分期在肺癌诊疗中的区别

Comparison level	TNM staging	TNMB staging
Core basis	Anatomical features	Anatomical features+hematological molecular markers
Assessment dimension	Static	Dynamic
Risk stratification	Difficult to identify biological heterogeneity within the same stage	It can refine subgroups to accurately identify high-risk patients
Prognostic accuracy	Limited differentiation among patients within the same stage	Better consistency and differentiation

### 3.1 分期核心依据

传统TNM分期的核心依据是肿瘤的解剖学特征，其本质是通过对T、N、M等解剖学指标的评估，界定疾病的进展程度，虽能在一定程度上反映肿瘤的发展阶段，但无法触及肿瘤的生物学本质。而TNMB分期则在保留TNM分期解剖学基础的同时，将血液中的分子标志物纳入评估体系，实现了解剖学特征与生物学特征的整合。这些分子标志物包括ctDNA、特定基因表达谱（如14基因分子预后分类器）等^[8,10,13]^，它们能够从基因、蛋白及代谢水平揭示肿瘤的增殖活性、侵袭潜能、耐药风险等生物学特性，使分期依据从单一的解剖学维度拓展至多维度的综合评估。

### 3.2 评估纬度

传统TNM分期主要依赖初始诊断时的影像学检查、手术标本病理分析等手段获取信息，其评估结果是静态的，难以反映肿瘤在疾病进展过程中的动态变化^[16]^。即使通过定期复查进行再评估，也无法实时捕捉治疗过程中肿瘤生物学行为的改变，导致对治疗反应的判断存在滞后性，有研究^[17]^显示，在NSCLC患者中生物标志物平均出现异常改变4.7个月后，提示疾病复发的影像学特征才会显现。TNMB分期则凭借血液分子标志物的可检测性，具备了动态监测的能力。例如，基于ctDNA的MRD检测可在治疗过程中实时追踪肿瘤残留情况，其动态变化（如从阳性转为阴性或持续阳性）能及时反映治疗效果及复发风险^[10]^。这种动态评估特性，使得TNMB分期能够更精准地捕捉肿瘤的进化轨迹，为调整治疗策略提供及时依据。

### 3.3 临床效能

在风险分层方面，传统TNM分期难以识别同一解剖学分期中存在的生物学异质性，导致部分患者被归入同一分期但实际预后差异显著。已有研究^[18]^表明，病理性IA期患者的5年OS率范围相对较广（77.0%-94.4%）。接受相同治疗的IA期肺腺癌患者也有不同的结果（复发、转移和死亡），而这差异与癌症组织学类型显著相关。TNMB分期通过分子标志物的补充，可将传统分期中“同质”的患者进一步细分为不同风险亚组，尤其是能更精准地识别高危患者^[8]^，为“风险适配”治疗策略的制定奠定基础。

在预后预测方面，传统TNM分期虽能对患者的生存情况进行大致区分，但区分效能有限。在一项针对第8版肺癌TNM分期系统的外部验证研究^[19]^中，IIIB、IIIC及IVA期患者的生存数据显示出明显的“重叠”现象：三者中位OS分别为9、8和6个月，5年OS率依次为9.2%、8.8%和5.5%。理想的分期系统应呈现随解剖学进展“生存时间逐步缩短”的阶梯式差异，但上述数据的实际差异幅度远小于预期。而TNMB分期通过整合分子标志物，显著提升了预后预测的精准度。

## 4 B分期血液分子标志物

第9版TNM分期虽强调分子标志物的重要性，但B分期尚未系统整合入TNM分期系统。通过明确已有研究中聚焦的分子标志物，能够为后续标准化研究提供依据。

### 4.1 B分期涉及的分子标志物

Lin的团队^[13]^的研究通过使用520或196基因的癌症相关panel开展NGS，调查发现肿瘤蛋白p53（tumor protein p53, *TP53*）、神经纤维蛋白1（neurofibromin 1, *NF1*）、细胞周期检测点激酶2（checkpoint kinase 2, *CHEK2*）、Kelch样ECH关联蛋白1（Kelch like ECH associated protein 1, *KEAP1*）、PTEN、视网膜母细胞瘤相关蛋白（retinoblastoma-associated protein, *RB1*）、磷脂酰肌醇-4,5-二磷酸3-激酶催化亚单位α（phosphatidylinositol-4,5-bisphosphate 3-kinase catalytic subunit alpha, *PIK3CA*）、低密度脂蛋白受体相关蛋白1B（low-density lipoprotein receptor-related protein 1B, *LRP1B*）、连环蛋白β1（catenin beta 1, *CTNNB1*）、肝细胞生长因子（hepatocyte growth factor, *HGF*）、腺瘤性息肉病基因/蛋白（adenomatous polyposis coli, *APC*）等基因突变与病理侵袭特征显著相关；表皮生长因子受体（epidermal growth factor receptor, *EGFR*）突变（尤其L858R）与低侵袭性特征[如LV阴性、信号转导衔接蛋白（signal transducing adaptor protein, STAS）阴性、1/2级]有关；间变性淋巴瘤激酶（anaplastic lymphoma kinase, *ALK*）、c-ros肉瘤致癌因子-受体酪氨酸激酶（ROS proto-oncogene 1, receptor tyrosine kinase, *ROS1*）融合与信号转导衔接蛋白（signal transducing adaptor protein, STAS）阳性、Grade 3级有关；*KRAS*、*BRAF *V600E仅与Grade 3级有关。

Kratz等^[8]^采用逆转录-定量聚合酶链式反应（reverse transcription quantitative polymerase chain reaction, RT-qPCR）检测14基因表达谱中各基因的表达水平，具体包括BCL2相关永生基因1（BCL2-associated athanogene 1, *BAG1*）、乳腺癌1号基因（breast cancer 1, early onset, *BRCA1*）、细胞分裂周期6（cell division cycle 6, *CDC6*）、细胞周期蛋白依赖性激酶2相关蛋白1（CDK2 associated protein 1, *CDK2AP1*）、Erb-B2受体酪氨酸激酶3（Erb-B2 receptor tyrosine kinase 3, *ERBB3*）、岩藻糖转移酶3（fucosyltransferase 3, *FUT3*）、白细胞介素11（interleukin 11, *IL-11*）、LCK原癌基因（LCK proto-oncogene, src family tyrosine kinase, *LCK*）、RND家族成员3（RND family member 3, *RND3*）、SH3结构域结合谷氨酸富含蛋白（SH3 domain binding glutamic acid-rich protein, *SH3BGR*）、Wnt 家族成员3A（Wnt family member 3A, *WNT3A*）、酯酶D（esterase D, *ESD*）、TATA盒结合蛋白（TATA-box binding protein, *TBP*）、Yes相关蛋白1（Yes-associated protein 1, *YAP1*）。测量后计算患者的风险水平评分，将患者分为低、中、高风险，用于TNMB分期中的“B”因子调整。该团队的第二项相关研究^[9]^采用的是相同的分子标志物和检测方法。

上述两个团队所研究的分子标志物来源于肿瘤组织，而在Chen等的研究^[10]^中，他们基于术后血浆样本中是否检测到分子残留病灶，即ctDNA，来判断患者体内是否还有残留的肿瘤细胞。利用个性化PROPHET技术，通过WES分析患者原发肿瘤组织的突变谱，筛选患者肿瘤细胞基因的特异性突变位点（每名患者最多筛选出50个），构建个性化检测panel。该技术结合独特分子标识符（unique molecular identifier, UMI）与超深度靶向测序，实现对血浆中ctDNA的高灵敏度检测。根据检测结果将患者分为MRD阳性（Blood 1, B1）或MRD阴性（Blood 0, B0），并与传统TNM分期系统结合，构建TNMB分期体系（如TNMB I、TNMB II等），用于更精准地评估NSCLC患者的术后复发风险和预后（表4^[8-10,13]^）。

**表4:** 4项研究中TNMB分期系统所采用的分子标志物与检测方法对比

Research	Source of molecular biomarkers	Type of markers	Detection methods	Definition of “B”
Genomic profiling of aggressive pathologic features in lung adenocarcinoma^[13]^	Tumor tissue	DNA mutation (*TP53, NF1, EGFR, ALK, etc*.）	NGS panel	Combining mutation profiles with pathological features
Incorporation of a molecular prognostic classifier improves conventional NSCLC staging^[8]^	Tumor tissue	mRNA expression (14 genes)	RT-qPCR	Risk score (Low/Medium/High)
Comparison of conventional TNM and novel TNMB staging systems for NSCLC^[9]^	Tumor tissue	mRNA expression (14 genes)	RT-qPCR	Ibid., external validation
Individualized tumor-informed ctDNA analysis for postoperative monitoring of NSCLC^[10]^	Plasma ctDNA	Personalized mutations (50 sites)	WES+UMI-deep sequencing	MRD status (B0/B1)

TP53: tumor protein 53; NF1: neurofibromin 1; EGFR: epidermal growth factor receptor; ALK: anaplastic lymphoma kinase; mRNA: messenger RNA; RT-qPCR: reverse transcription quantitative polymerase chain reaction; WES: whole exome sequencing; UMI: unique molecular identifier.

### 4.2 其他常见分子标志物

目前许多肺癌研究虽未明确提及TNMB分期，但却聚焦于影响肺癌诊疗的分子标志物。比如，基因表达谱通过分析肿瘤中基因的活跃程度，能更细致地进行肿瘤亚型分析，对有靶向基因突变的患者，基因表达谱可进一步分类，辅助判断患者接受靶向治疗的获益情况并解释治疗反应差异^[20]^；而免疫组化标志物可辅助肺癌精准诊断和治疗决策，一方面，即使在活检样本少、肿瘤分化程度低的情况下，通过甲状腺转录因子1（thyroid transcription factor 1, TTF-1）、P40等特定标志物也可精准区分肿瘤亚型，另一方面，其可为靶向治疗和免疫治疗提供支持^[21]^。

下面通过检索相关研究，对目前常见的分子标志物进行梳理，期望为后续B分期共识形成提供一定参考（表5）。在Pubmed中通过检索式[“lung neoplasms” (MeSH Major Topic)] AND {“biomarkers” (MeSH Major Topic) OR “immunohistochemistry” (MeSH Major Topic) OR [“mutant” (All Fields) OR “mutants” (All Fields) OR “mutants” (All Fields)]} AND {“genes” (MeSH Major Topic) OR [“genes” (MeSH Terms) OR “genes” (All Fields) OR “gene” (All Fields)]} AND [“diagnose” (Title/Abstract) OR “staging” (Title/Abstract) OR “stage” (Title/Abstract)] AND [y_5(Filter)] AND [randomized controlled trial (Filter) OR systematic review (Filter)]展开检索，过滤器设置为近5年的系统综述及随机对照研究，共查询到24篇文章。纳入研究肺癌诊断、分期及预后的研究，排除（1）利用已知的生物分子靶点开展的研究；（2）治疗性研究；（3）其余内容严重不符的研究。共排除17篇文章，纳入其余7篇文献^[22-28]^。在Web of Science中，通过检索式{[lung cancer (topic)] and [biomarker OR gene OR antigen OR immunohistochemistry (topic)] and [diagnose OR stage (topic)]}展开检索，选择高被引、近5年的Article和Review article，共查询到68篇文章。依据类似的纳排标准，同时排除非系统综述文章和PubMed检索中纳入的文章后，共排除63篇文章，纳入余下5篇文献^[29-33]^。

**表5:** 肺癌分子标志物汇总

Category	Molecular biomarkers
Gene mutation^[22]^	*TP53, EGFR, KRAS, PIK3CA, BRAF, MET, ALK, ROS1, RET, ERBB2*
DNA methylation^[23]^	*SHOX2, SOX17, HOXA9, SEPT9, RASSF1A, PTGER4, APC*
miRNA^[24]^	miR-21, miR-210, miR-486-5p, miR-126, miR-375
Tumor-associated protein^[25]^	CEA, CYFRA21-1, Pro-GRP
Others	circRNAs^[26]^, *ALDH1*^[27]^, *NM23*^[28]^, *CDCA8*^[29]^, CTC, ctDNA^[30,31]^, EV, tumor metabolites, TEP, TAA^[32,33]^

KRAS: Kirsten rat sarcoma viral oncogene homolog; PIK3CA: phosphatidylinositol-4,5-bisphosphate 3-kinase catalytic subunit alpha; BRAF: B-Raf proto-oncogene, serine/threonine kinase; MET: mesenchymal-epithelial transition factor; ROS1: ROS proto-oncogene 1, receptor tyrosine kinase; RET: rearranged during transfection; ERBB2: human epidermal growth factor receptor 2; SHOX2: short stature homeobox 2; SOX17: transcription factor SOX-17; HOXA9: homeobox A9; SEPT9: septin 9; RASSF1A: ras association domain family member 1, isoform A; PTGER4: prostaglandin E receptor 4; APC: adenomatous polyposis coli; miR-21: microRNA-21; miR-210: microRNA-210; miR-486-5p: microRNA-486-5p; miR-126: microRNA-126; miR-375: microRNA-375; CEA: carcinoembryonic antigen; CYFRA21-1: cytokeratin 19 fragment 21-1; Pro-GRP: pro-gastrin-releasing peptide; circRNAs1: circular RNAs 1; ALDH16: aldehyde dehydrogenase 16 family member A1; NM23: non-metastatic protein 23; CDCA8: cell division cycle associated 8; CTC: circulating tumor cell; EV: extracellular vesicles; TEP: tumor-educated platelets; TAA: tumor-associated antigen.

检索得到的相关分子标志物在肺癌诊疗中均展现出了各自独特的价值。比如，在诊断与分型方面，血液中的*miR-20a*、*miR-10b*、*miR-150*和*miR-223*对NSCLC具有优异的诊断价值，灵敏度和特异性均超过0.8，而*miR-205*是鳞癌的特异性标志物，能有效区分鳞癌与腺癌等其他亚型，在预后预测中，*miR-34a*、*miR-93*、*miR-106*等标志物的表达水平与免疫检查点抑制剂治疗响应有关，可提前预判患者对免疫治疗的获益情况；ctDNA作为液体活检的核心标志物，术前检测可辅助区分肺腺癌与非腺癌亚型，非腺癌患者的ctDNA阳性率（78/85）显著高于肺腺癌（39/93），且术前检测阳性的腺癌患者更易出现临床隐匿性纵隔淋巴结转移，可为病理分期补充分子层面的依据。除上述描述的miRNA和ctDNA外，DNA甲基化相关分子、肿瘤相关蛋白等分子标志物在肺癌诊疗中也提供了重要补充（表5）。

这些分子标志物从不同维度揭示了肺癌的生物学特征，未来TNMB分期有必要全面纳入血液受累、基因突变、免疫组化等多方面的分子参数，以期对肺癌患者进行更全面的分期、分层，为患者的预后判断和治疗决策提供更坚实的依据。

## 5 小结与展望

TNMB分期在传统TNM分期基础上整合血液分子标志物，实现了解剖学与生物学特征的多维度评估。研究^[8-10,13]^表明，其在肺癌风险分层中可精准识别高危患者，净重新分类改善显著；预后预测准确性更高，能避免传统分期生存数据重叠问题；且可动态监测治疗反应与复发风险，为临床决策提供可靠依据，在诊断、治疗及预后评估中优势明显。

目前，对TNMB分期的研究亦具有一定的局限性：（1）分子标志物缺乏标准化，检测方法与阈值未统一，由于TNMB分期在肺癌领域应用时间短，肺癌进行B分期时应该选用何种血液分子标志物尚未形成共识；不同研究选用不同的分子标志物，哪些具有治疗和预后价值暂时不能确认；且缺乏大规模、多中心、前瞻性的验证研究支持其在不同人群中的适用性。（2）研究多集中于NSCLC，需在亚型中进一步验证，未来需通过多中心研究确立核心标志物及标准流程，扩展应用范围。（3）B分期依赖分子及基因检测技术，这些检测技术在不同地区的可行性及准确性存在较大差异，对于未来的普及有较大的阻碍。作为一项全新的分期，B分期要如何与TNM分期整合互补，它是否和TNM分期占有相似的权重，整合后分期需要如何调整，都是亟待解决的问题。

随着分子生物学技术的不断发展和临床研究的深入，TNMB分期有望逐步完善并广泛应用于临床，推动肺癌诊疗从“解剖学导向”向“解剖+生物学特征导向”的精准化模式迈进，为实现个体化治疗提供更坚实的理论与实践支撑。

## References

[1] Yao YF, Sun KX, Zheng RS. Interpretation and analysis of the Global Cancer Statistics Report 2022:a comparisonbetween China and the world. Zhongguo Puwai Jichu Yu Linchuang Zazhi, 2024, 31(7): 769-780.

[2] Carbone DP, Hirsch FR, Osarogiagbon RU, et al. The International Association for the Study of Lung Cancer staging project: The impact of common molecular alterations on overall survival in NSCLC ininitial analyses of the IASLC ninth edition staging database. J Thorac Oncol, 2025, 20(10): 1423-1440. doi: 10.1016/j.jtho.2025.06.025 40920144

[3] Mountain CF. Revisions in the international system for staging lung cancer. Chest, 1997, 111(6): 1710-1717. doi: 10.1378/chest.111.6.1710 9187198

[4] Liu BD, Zhi XY. Interpretation and prospect of the eighth edition of TNM staging for lung cancer. Shoudu Yike Daxue Xuebao, 2016, 37(6): 753-757.

[5] Zhang B, Fang WT, Zhong H. Introduction to the 9^th^ edition of TNM classification for lung cancer. Zhongguo Zhongliu Zazhi, 2024, 46(3): 206-210. 10.3760/cma.j.cn112152-20231017-00203 38494767

[6] Rami-Porta R, Nishimura KK, Giroux DJ, et al. The International Association for the Study of Lung Cancer lung cancer staging project: Proposals for revision of the TNM stage groups in the forthcoming (ninth) edition of the TNM classification for lung cancer. J Thorac Oncol, 2024, 19(7): 1007-1027. doi: 10.1016/j.jtho.2024.02.011 38447919

[7] Yang M, Forbes ME, Bitting RL, et al. Incorporating blood-based liquid biopsy information into cancer staging: time for a TNMB system?. Ann Oncol, 2018, 29(2): 311-323. doi: 10.1093/annonc/mdx766 29216340 PMC5834142

[8] Kratz JR, Haro GJ, Cook NR, et al. Incorporation of a molecular prognostic classifier improves conventional non-small cell lung cancer staging. J Thorac Oncol, 2019, 14(7): 1223-1232. doi: 10.1016/j.jtho.2019.03.015 30959120

[9] Haro GJ, Sheu B, Cook NR, et al. Comparison of conventional TNM and novel TNMB staging systems for non-small cell lung cancer. JAMA Netw Open, 2019, 2(12): e1917062. doi: 10.1001/jamanetworkopen.2019.17062 PMC6902768 31808928

[10] Chen K, Yang F, Shen H, et al. Individualized tumor-informed circulating tumor DNA analysis for postoperative monitoring of non-small cell lung cancer. Cancer Cell, 2023, 41(10): 1749-1762.e6. doi: 10.1016/j.ccell.2023.08.010 37683638

[11] Chen Z, Lin Y, Qin Y, et al. Prognostic factors and survival outcomes among patients with mycosis fungoides in China: A 12-year review. JAMA Dermatol, 2023, 159(10): 1059-1067. doi: 10.1001/jamadermatol.2023.2634 37585188 PMC10433139

[12] Hristov AC, Tejasvi T, Wilcox RA. Cutaneous T-cell lymphomas: 2023 update on diagnosis, risk-stratification, and management. Am J Hematol, 2023, 98(1): 193-209. doi: 10.1002/ajh.26760 36226409 PMC9772153

[13] Lin YD, Li HJ, Hong HZ, et al. Genomic profiling of aggressive pathologic features in lung adenocarcinoma. Lung Cancer, 2025, 203: 108460. doi: 10.1016/j.lungcan.2025.108460 40179539

[14] Kratz JR, He J, Van Den Eeden SK, et al. A practical molecular assay to predict survival in resected non-squamous, non-small-cell lung cancer: Development and international validation studies. Lancet, 2012, 379(9818): 823-832. doi: 10.1016/S0140-6736(11)61941-7 22285053 PMC3294002

[15] Zhang JT, Liu SY, Gao W, et al. Longitudinal undetectable molecular residual disease defines potentially cured population in localized non-small cell lung cancer. Cancer Discov, 2022, 12(7): 1690-1701. doi: 10.1158/2159-8290.CD-21-1486 35543554 PMC9394392

[16] Lim ZF, Ma PC. Emerging insights of tumor heterogeneity and drug resistance mechanisms in lung cancer targeted therapy. J Hematol Oncol, 2019, 12(1): 134. doi: 10.1186/s13045-019-0818-2 PMC6902404 31815659

[17] Herbst RS, John T, Grohé C, et al. Molecular residual disease analysis of adjuvant osimertinib in resected *EGFR*-mutated stage IB-IIIA non-small-cell lung cancer. Nat Med, 2025, 31(6): 1958-1968. doi: 10.1038/s41591-025-03577-y 40097663 PMC12176615

[18] Zhang J, Wu J, Tan Q, et al. Why do pathological stage IA lung adenocarcinomas vary from prognosis?: A clinicopathologic study of 176 patients with pathological stage IA lung adenocarcinoma based on the IASLC/ATS/ERS classification. J Thorac Oncol, 2013, 8(9): 1196-1202. doi: 10.1097/JTO.0b013e31829f09a7 23945388

[19] Hwang JK, Page BJ, Flynn D, et al. Validation of the eighth edition TNM lung cancer staging system. J Thorac Oncol, 2020, 15(4): 649-654. doi: 10.1016/j.jtho.2019.11.030 31863848

[20] Hijazo-Pechero S, Alay A, Marín R, et al. Gene expression profiling as a potential tool for precision oncology in non-small cell lung cancer. Cancers (Basel), 2021, 13(19): 4734. doi: 10.3390/cancers13194734 PMC8507534 34638221

[21] Osmani L, Askin F, Gabrielson E, et al. Current WHO guidelines and the critical role of immunohistochemical markers in the subclassification of non-small cell lung carcinoma (NSCLC): Moving from targeted therapy to immunotherapy. Semin Cancer Biol, 2018, 52 (Pt 1): 103-109. doi: 10.1016/j.semcancer.2017.11.019 29183778 PMC5970946

[22] Parra-Medina R, Castañeda-González JP, Montoya L, et al. Prevalence of oncogenic driver mutations in hispanics/latin patients with lung cancer. A systematic review and *meta*-analysis. Lung Cancer, 2023, 185: 107378. doi: 10.1016/j.lungcan.2023.107378 37729688

[23] Machado DI, Brito-Rocha T, Salta S, et al. Circulating cell-free DNA methylation as biomarker for lung cancer detection: A systematic review and *meta*-analysis of diagnostic studies. Syst Rev, 2025, 14(1): 120. doi: 10.1186/s13643-025-02860-w PMC12128315 40457503

[24] Zhong S, Golpon H, Zardo P, et al. miRNAs in lung cancer. A systematic review identifies predictive and prognostic miRNA candidates for precision medicine in lung cancer. Transl Res, 2021, 230: 164-196. doi: 10.1016/j.trsl.2020.11.012 33253979

[25] Gan T, An W, Long Y, et al. Correlation between carcinoembryonic antigen (CEA) expression and *EGFR* mutations in non-small cell lung cancer: a *meta*-analysis. Clin Transl Oncol, 2024, 26(4): 991-1000. doi: 10.1007/s12094-023-03339-7 38030870

[26] Zheng Y, Hu J, Li Y, et al. Clinicopathological and prognostic significance of circRNAs in lung cancer: A systematic review and *meta*-analysis. Medicine (Baltimore), 2021, 100(14): e25415. doi: 10.1097/MD.0000000000025415 PMC8036086 33832139

[27] Li D, Cao Y, Luo CW, et al. The clinical significance and prognostic value of ALDH 1 expression in non-small cell lung cancer: A systematic review and *meta*-analysis. Recent Pat Anticancer Drug Discov, 2024, 19(5): 599-609. doi: 10.2174/0115748928265992230925053308 37818578

[28] Min SH, Zheng QQ. Clinicopathological and prognostic significance of NM23 expression in patients with non-small cell lung cancer: A systematic review and *meta*-analysis. Medicine (Baltimore), 2021, 100(47): e27919. doi: 10.1097/MD.0000000000027919 PMC8615335 34964763

[29] Hu H, Umair M, Khan SA, et al. CDCA8, a mitosis-related gene, as a prospective pan-cancer biomarker: Implications for survival prognosis and oncogenic immunology. Am J Transl Res, 2024, 16(2): 432-445. doi: 10.62347/WSEF7878 38463578 PMC10918119

[30] Abbosh C, Frankell AM, Harrison T, et al. Tracking early lung cancer metastatic dissemination in TRACERx using ctDNA. Nature, 2023, 616(7957): 553-562. doi: 10.1038/s41586-023-05776-4 37055640 PMC7614605

[31] Xia L, Mei J, Kang R, et al. Perioperative ctDNA-based molecular residual disease detection for non-small cell lung cancer: A prospective multicenter cohort study (LUNGCA-1). Clin Cancer Res, 2022, 28(15): 3308-3317. doi: 10.1158/1078-0432.CCR-21-3044 34844976

[32] Lone SN, Nisar S, Masoodi T, et al. Liquid biopsy: A step closer to transform diagnosis, prognosis and future of cancer treatments. Mol Cancer, 2022, 21(1): 79. doi: 10.1186/s12943-022-01543-7 PMC8932066 35303879

[33] Ren F, Fei Q, Qiu K, et al. Liquid biopsy techniques and lung cancer: diagnosis, monitoring and evaluation. J Exp Clin Cancer Res, 2024, 43(1): 96. doi: 10.1186/s13046-024-03026-7 PMC10985944 38561776

